# New Inventions

**Published:** 1897-09

**Authors:** 


### NEW INVENTIONS.

A ball-float, the lever provided with a series of rods or arms of spring-metal, projecting from the lever in different radial directions, curved downwardly, and extended to embrace and confine with a constrictive pressure; the holder of the ball consisting of an inverted cup-shape casting, having an upwardly extended, transversely apertured hub; the edgewise arranged bosses provided with screw sockets.

A horse-cover with a pair of pivoted arms secured to the edges of the neck portion of the blanket, and mechanism secured to the blanket for turning said arms on their pivots.

An automatic cattle-gate for railways, comprising a bearing-block secured to the bottom flanges of the rails, a cam-faced shaft provided with cylindrical journals, the gate-panels and radial arm fixed to said shaft, the weight secured to the arm by the chain, in combination with the platforms, pivoted at one end to the fixed shaft, a chain connecting the shaft and the free ends of the platforms.

The combination with a horseshoe having a depending ledge upon its under side, provided with dovetailed recesses, of a plate fitting with the shoe; lugs upon the upper surface of plate having devotailed recesses registering with the recesses in the ledge; detachable calks having laterally extending dovetailed tongues adapted to fit within registering recesses, and screws passing through the tongues and lugs.

A horse-checking device operated from the seat of wagon through rods whose ends are arranged to drop in teeth arranged around the hub.

An improved hoof-pad, consisting of two parts of metal interlocking in the centre over the point of the frog.

An ice-creeper shell, carrying calks, and provided near its rear end with an opening through which the heel-calk of the shoe is adapted to pass.

A harness-saddle, comprising a saddle-seat, stems projecting downwardly therefrom, crupper-loop, a skirt for supporting the crupper-loop, and a bearing-strap adapted to move between the saddle-seat and crupper-loop and be guided by the stems.

An apparatus for aligning and starting horses on race-tracks, consisting of a spring-stretched, flexible barrier, having its ends connected to spring-raised brackets, movable in slotted columns, and levers adapted to engage teeth on said brackets, and connected together through bell-cranks pivoted on opposite sides of the track.

A horseshoe with curved calk projecting from lower face of the toe of said body, and an independent L-shaped calk projecting from the lower face of each of the heels; has long edges parallel with the outer edge, and the several calks merge nearby the centre of the shoe.

A checkrein-hook, combination with a post of a hook, the shank being swivelled to the post and provided with a transverse hoop which is adapted to be attached to the crupper-strap of the harness.

A currycomb with rows of teeth connected to back, a cleaning plate having openings through which the teeth can pass; the shoulders serve as abutments for the back, the coil springs are located between the back and the cleaning plate.

A line or rein-guide or holder, adapted to be connected with the hip-strap of the harness.

A holder for powder-papers, consisting of a base, the guide-posts, the pivoted latch at the upper end of one of the guide-posts, and the spring-actuated table arranged to slide on posts between the nose of the latch and the base.

A veterinary tooth-cutter, composed of parallel roller-bars, formed with sector-gears, each of the bars being provided at one end with a handle and at the other with a cutting-edge and connected by bearing-boxes.

A bridle-bit, the combination with the bit-bar having apertured ends that taper inward from opposite sides of the rein-rings, having opposite conical ends received into the tapering apertured ends of the bit-bar, and means for drawing said conical ends toward each other to tighten them in the bit.

A harness-buckle, the plate having orifices near the ends and the bend, the plate provided with slot, spring, and peg, in combination with a movable hand.

A clinical thermometer, having two distinct chambers formed within the bore of the tube, and a comparatively open contraction between both chambers.

A bandage-cutting machine, with knives and transversely slotted rests adjacent thereto.

A hame-body, formed from sheet-metal blank, condensing the metal at the place of the greatest wear and tear and permitting the body to be formed from a comparatively thin blank.

A cauterizing apparatus, in combination with a burner-tube of a blower-holder, carried by the handle.

A soft-tread horseshoe, the groove tread and band for retaining tread in position, and having its ends held in the groove in the tread.

An equalizer for preventing horses from leaving their feet, the combination with boots of a cord connecting the boots and a guide adapted to be secured against the under side of the body, and held against forward movement.

